# The emerging role of the piRNA/PIWI complex in respiratory tract diseases

**DOI:** 10.1186/s12931-023-02367-9

**Published:** 2023-03-13

**Authors:** Yizhu Yao, Yaozhe Li, Xiayan Zhu, Chengguang Zhao, Lehe Yang, Xiaoying Huang, Liangxing Wang

**Affiliations:** 1 Division of Pulmonary Medicine, The First Affiliated Hospital, Wenzhou Medical University, Wenzhou, 325000 Zhejiang China; 2 School of Pharmaceutical Sciences, Wenzhou Medical University, Wenzhou, 325035 Zhejiang China

**Keywords:** Non-coding RNAs, PIWI protein, PIWI-interacting RNA, Respiratory tract diseases

## Abstract

PIWI-interacting RNA (piRNA) is a class of recently discovered small non-coding RNA molecules with a length of 18–33 nt that interacts with the PIWI protein to form the piRNA/PIWI complex. The PIWI family is a subfamily of Argonaute (AGO) proteins that also contain the AGO family which bind to microRNA (miRNA). Recently studies indicate that piRNAs are not specific to in the mammalian germline, they are also expressed in a tissue-specific manner in a variety of human tissues and participated in various of diseases, such as cardiovascular, neurological, and urinary tract diseases, and are especially prevalent in malignant tumors in these systems. However, the functions and abnormal expression of piRNAs in respiratory tract diseases and their underlying mechanisms remain incompletely understood. In this review, we discuss current studies summarizing the biogenetic processes, functions, and emerging roles of piRNAs in respiratory tract diseases, providing a reference value for future piRNA research.

## Background

Noncoding RNA (ncRNA) is a group of RNA molecules that are transcribed but do not encode proteins [1]. PIWI-interacting RNA (piRNA) is a class of small noncoding RNA (sncRNA) [2]. The first piRNA was discovered in 2001 in *Drosophila* testes by Aravin as a small RNA derived from the Su (Ste) tandem repeats, which silence transcripts to maintain male fertility [3]. The small RNA was named piRNA until they were separated from mice testes which guided for mammalian PIWI proteins in the male germ line [3]. Studies have shown that MIWI/MILI, a murine PIWI protein, binds a previously uncharacterized class of 26–30-nucleotide (nt) RNAs that are highly abundant in testes. To date, piRNAs have been comprehensively studied in other organisms such as arthropods, worms, humans and rats, in both germ cells and somatic cells [4–12]. Additional studies have shown that piRNAs are significantly different from other sncRNAs in their characteristics, biogenesis and functions. These differences are summarized as follows: (1) the size of piRNAs is approximately 18–33nt [13, 14]; (2) piRNAs originated from two types of piRNA clusters: the uni-stranded cluster and the dual-stranded cluster [1]; (3) piRNAs usually have 3ʹ-2-O-methylation [15–23]. While miRNA do not have any of those characteristics. In recent years, various piRNA-related high-throughput data have been collected and integrated into several databases including piRBase [24],piRNAQuest [25], and piRNABank [26], and the piRNA cluster database [27]. The piRBase currently lists 8,438,265 piRNAs in *Homo sapiens*. Moreover, recent studies have described a large number of piRNAs and their related PIWI protein expression in somatic cells, with some piRNA/PIWI complexes participating in numerous diseases, including respiratory tract disease [4, 28]. In this study, the formation, function and mechanism of piRNAs and the research progress on the relationship between piRNA/PIWI protein and respiratory tract disease in recent years are reviewed, which will provide a reference for further exploration of the mechanism of piRNAs.

## The biosynthesis of piRNAs

**Fig. 1:**
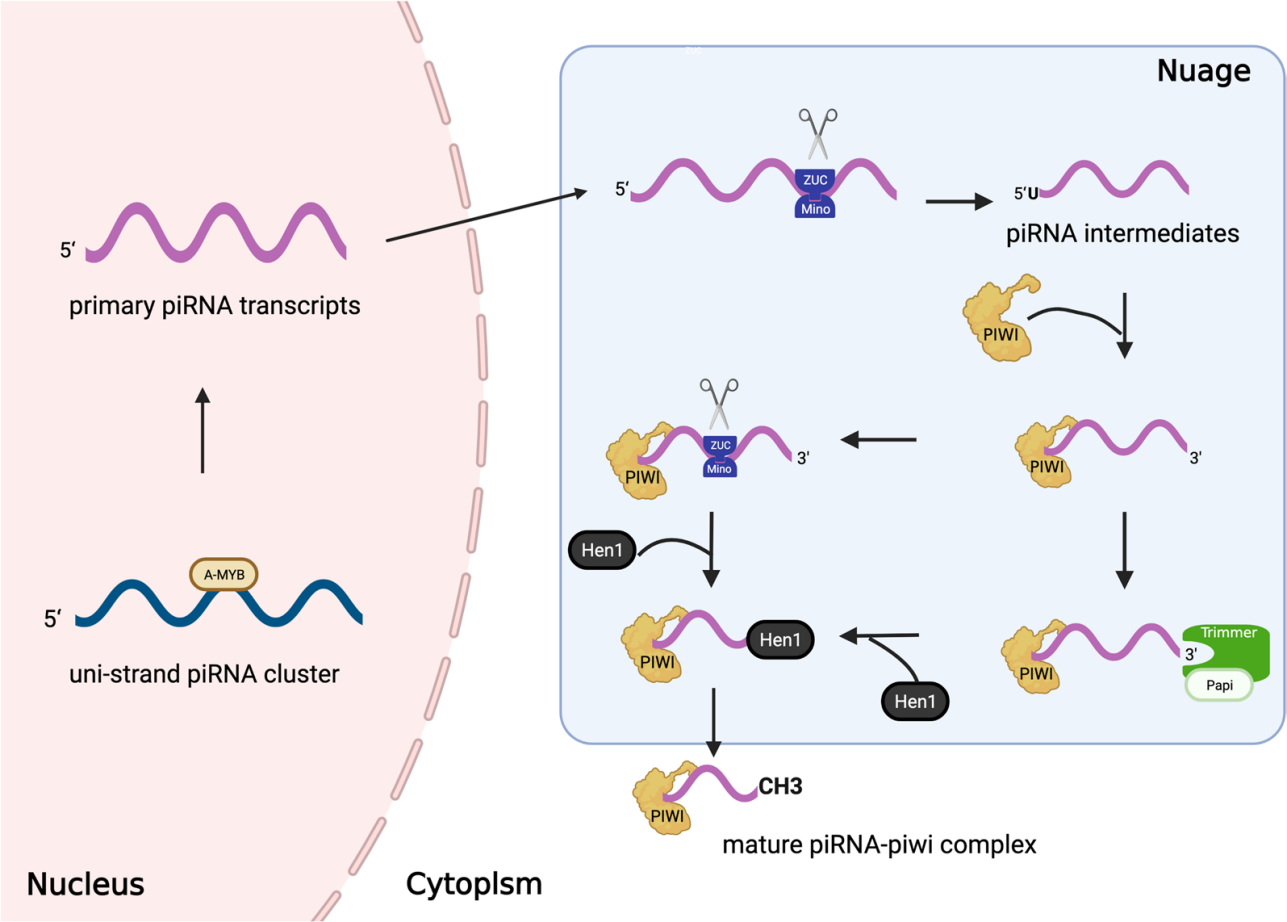
The biosynthesis of PIWI-interacting RNAs (PiRNAs). In the nucleus, the uni-stranded piRNA cluster are transcribed into the primary piRNAs transcripts, which are transported to the cytoplasmic nuage. There, the primary piRNAs transcripts are spliced by Zuc and its co-factor (mino) to produce piRNA intermediates with 5ʹuracil. After binding to the PIWI protein, the 3ʹ end is modified by Zuc or Trimmer and its cofactor Papi, which is an exonuclease. Following methylation by Hen1, the mature piRNA-PIWI complex is generated

**Fig. 2:**
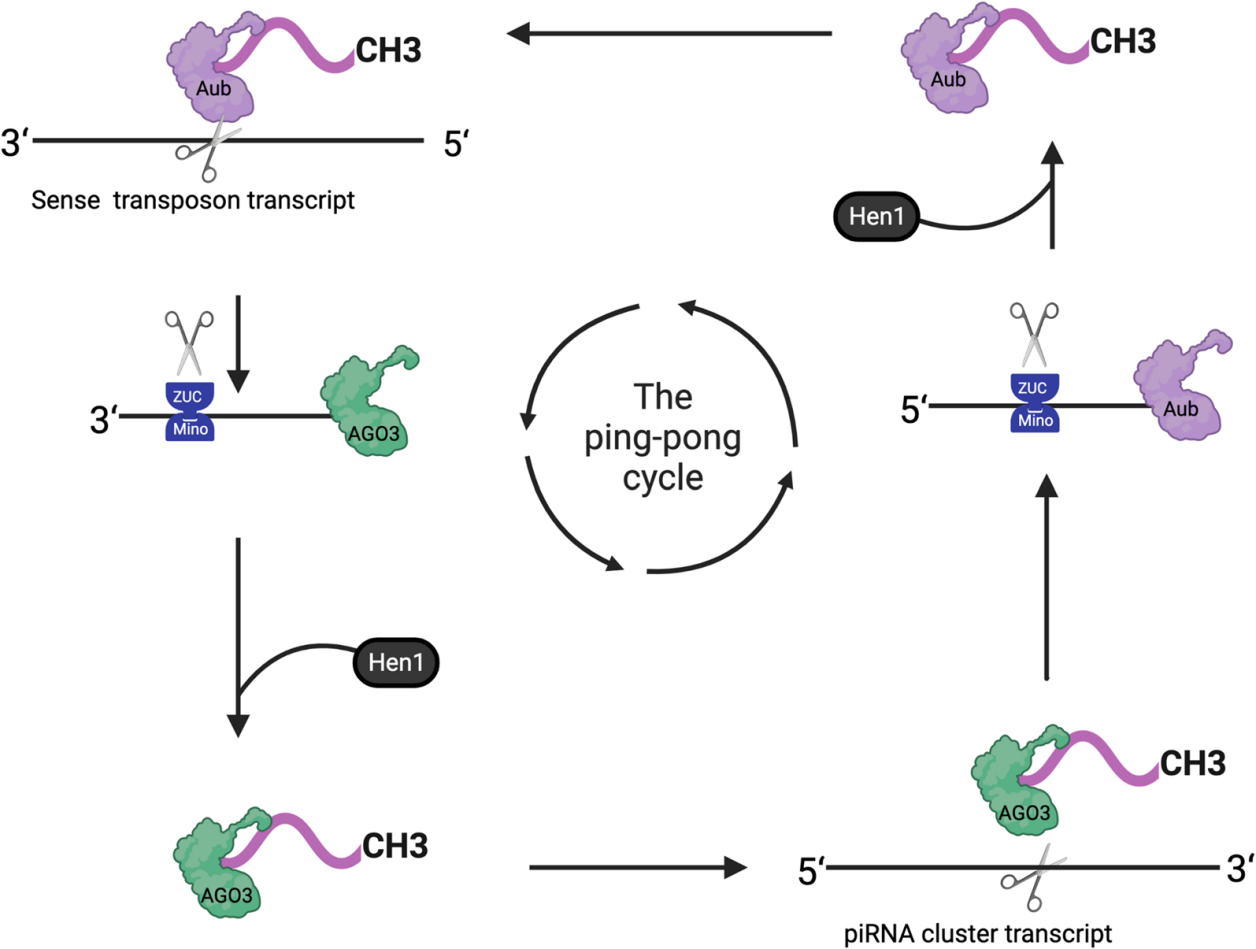
The ping-pong cycle. The antisense piRNA (5ʹ-3ʹ) binding with the Aub protein cuts the transcript of the transposon mRNA to produce sense piRNA (3ʹ-5ʹ). Then, the sense piRNA (3ʹ-5ʹ) binds with the AGO3 protein and becomes mature sense piRNAs through shear and methylation in in a similar manner. The mature sense piRNAs (3ʹ-5ʹ) combine with the piRNA cluster transcript and sliced it to produce antisense piRNA (5ʹ-3ʹ), which is repeated in the ping-pong cycle. The piRNA produced in the ping-pong cycle is also called secondary piRNA

Mature piRNAs are generated by two distinct pathways: the primary maturation pathway, which is directly encoded from the piRNA cluster (Fig. 1), and the ping-pong cycle (Fig. 2). We call piRNAs produced from the latter route secondary piRNAs. Conceptually, primary piRNA biogenesis can be divided into several steps. The first step is the transcription of piRNA. A large fraction of piRNAs originate from two specific types of genomic loci, named the piRNA cluster [15–17, 29].The mammalian piRNA cluster which is a uni-stranded clusters contains a promoter element (A-MYB), RNA polymerase II and downstream components marked by histone 3 lysine 4 dimethylation (H3K4me3) [30–32]. After undergoing 5ʹ capping, 3ʹ polyadenylation and alternative splicing, the cluster eventually produce the piRNAs transcripts. In flies, the dual-stranded cluster has histone 3 lysine 9 dimethylation/trimethylation (H3K9me2/3) marks modifications and depends on promoters in neighboring coding genes to initiate transcription [18, 22, 31, 33–37]. However, most dual-strand clusters transcription is promoter-less and relies on proteins Rhino and Moonshiner [33]. Next, piRNAs transcripts are transported to the cytoplasmic nuage through nuclear pores and combined with Zucchini (ZUC) and its cofactors including Minotaur (Mino), Vreteno (Vert), and a leucine zipper (Gasz), which act as an endonuclease to modify the 5ʹend of pre-piRNAs, producing piRNA intermediates with 5ʹ uracil [38–52]. Finally, piRNA intermediates bind to the PAZ domain of the PIWI protein and recruit the Trimmer/PNLDC1 to modify the 3ʹend of the piRNA [48, 53, 54]. Subsequent methylation by Hen1 yields the mature piRNA-PIWI complex [22, 55, 56]. The ping-pong cycle plays a crucial role in the amplification of piRNAs through the piRNA-dependent post-transcriptional gene silencing (PTGS) mechanism [18, 22, 44, 57, 58]. Following the production of primary piRNAs, Aubergine (Aub)-bound antisense piRNA initiates the ping-pong cycle by splicing transposon mRNA transcripts and generating a sense-oriented piRNA intermediate. The sense piRNA intermediate is bound to the AGO3 protein and trimmed to produce mature sense piRNA (secondary piRNA). Similar to the steps described above, AGO3-bound sense piRNA can bind and cleave the antisense transposon sequences present in the transcripts of the original piRNA cluster, producing antisense piRNAs intermediates which then bind Aub protein. As a result, the cycle reinitiates [59, 60].

## PiRNA/PIWI complex function and mechanism in respiratory tract diseases

Recent studies indicate that piRNAs exist in a variety of tissues in multiple organisms and contribute to the physiological and pathological processes at the transcriptional or post-transcriptional level [61–63]. Here, we summarize the function and mechanism of piRNAs in respiratory tract diseases.

### PiRNA/PIWI complex-mediated transcriptional gene silencing (TGS)

PiRNA/PIWI complexes bound to the Asterix (Arx) protein enter the nucleus, and scan for nascent transposon transcription through complementary sequence [64, 65]. In drosophilids, after identification, the piRNA-PIWI/Arx complexes are combined with panoramix (Panx) and induce transcriptional gene silencing (TGS) by recruiting general silencing machinery components [30, 66]. As a result, repressive histone 3 lysine 9 trimethylation (H3K9me) marks are added in the target transposon, induced by Eggless (Egg) and its cofactor Windei (Wde), leading to heterochromatin protein 1 (HP1) recruitment and subsequently heterochromation formation [31, 67–69]. In mammals, SPOCD1 is bound to MIWI2 and participated in young transposon methylation and silencing. SPOCD1 co-purified in vivo with DNMT3L and DNMT3A, components of the de novo methylation machinery [70]. One study used reduced representation bisulfite sequencing (RRBS) to perform global DNA methylation analyses and found that the RASSF1-PIWI1-piRNA pathway could modulate key oncogenes and tumor suppressor genes by methylated Gen interacting protein (GMIP) [71]. PiRNA-overexpression facilitated the DNA methylation of the acly-CoA dehydrogenase (Acadm) promoter region, repressing Acadm expression and promoting pulmonary arterial smooth muscle cell (PASMC) proliferation [72].

### PiRNA/PIWI complex-mediated post-transcriptional gene silencing (PTGS)

There is a relationship between the mechanisms of miRNA silencing and PTGS of piRNA/PIWI complexes. Mature miRNAs combined with the RNA-induced silencing complex (RISC) silence gene through translation repression and mRNA decay while piRNA-induced silencing complexes, consisting of PIWI protein, piRNAs, CAF1 deadenylase, occasionally recruit carbon catabolite-repressed 4-negative on TATA-less (CCR4-NOT) and Smaug (Smg), and mediate mRNA deadenylation and decay through an miRNA-similar mechanism [73]. The RNAs of piRNA-RNA interaction include mRNA, transcribed pseudogenes, and long noncoding RNA (lncRNA) [74–76]. Combined analyses of small RNA sequencing of peripheral blood collected from pulmonary tuberculosis (PTB) patients and healthy individuals demonstrated that one mRNA can be regulated by several piRNAs. From the constructed network of upregulated piRNAs and downregulated mRNAs, piRNA-881565, piRNA-489848, piRNA-1869760, piRNA-784007 and piRNA-1503138 regulated 34, 32, 28, 21 and 18mRNA targets, respectively [77]. Furthermore, piR-55490 binds the mTOR 3ʹ-UTR, inducing mRNA degradation and repression of lung cancer growth [78]. In radiation-induced lung fibrosis (RILF), Nrf2 signaling increased the expression of PIWI-like RNA-meditate gene silencing 2 (PIWIL2), which is usually upregulated in somatic cells during DNA damage to promote repair by remodeling chromatin [79]. Furthermore, by screening the bronchial smooth muscle (BSM) cell transcriptome for targets of the piRNAs differentially expressed in asthma samples, Elena Alexandrova et al. revealed that some mRNAs had multiple possible binding sites for the same piRNA, located in different domains of the molecule (5ʹ-UTR, coding DNA sequence, or 3ʹ-UTR), while others were complementary to two complete differentially expressed piRNAs. Interestingly, many pseudogenes and lncRNAs are also potential targets of asthma-specific piRNAs [80].

### PiRNA/PIWI complex-mediated protein modification

Some studies have shown that piRNAs and piRNA/PIWI complexes directly bind to some proteins, which is dependent on the piRNAs or the PAZ domain of the PIWI protein. The central part of the UUNNUUUNNUU motif in piRNA-like-163 (piR-L-163) directly interacted with the RRRKPDT element of phosphorylated ERM proteins, promoting the proliferation and migration in both human bronchial epithelial (HBE) cells and normal HBE (NHBE) cells [81]. Yuyan Wang et.al found that piRNA-L-138 bound the p60-MDM2 (mouse double minute 2 homolog) and inhibited chemoresistance to cisplatin (CDDP)-activated apoptosis in p53-mutated lung squamous cell carcinoma (LSCC) [82].

## PiRNA/PIWI complex in respiratory tract diseases

**Fig. 3:**
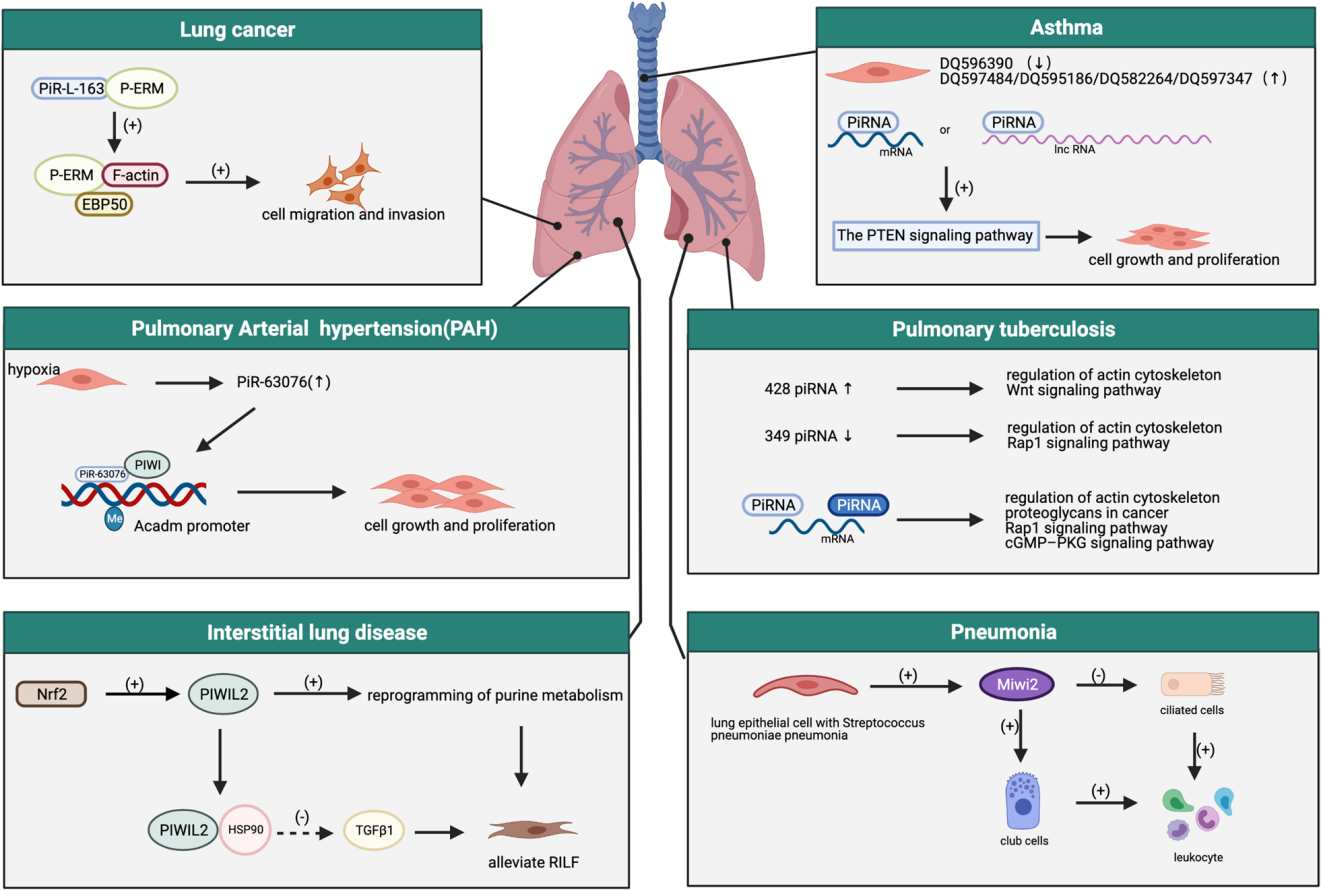
PiRNAs/PIWI complex as a diagnostic biomarker and therapeutic target in respiratory tract diseases. piRNA-L-163 directly bonded to p-ERM and regulated its activity, affecting the proliferation and migration ability of lung cancer cells. There are 5 piRNAs (DQ596390, DQ597484, DQ595186, DQ582264, DQ597347) differently expressed in BSM cells between asthmatic patients and healthy subjects, which plays a role in the development of asthma through the PTEN signaling pathway. piR-63076 regulated cell proliferation and proliferation by increasing the methylation status of the Acadm promoter. In the process of TB, there are 428 upregulated piRNAs and 349 downregulated piRNAs involved in regulation of the actin cytoskeleton, proteoglycans of cancer, the Rap1 signaling pathway and the cGMP–PKG signaling pathway. Moreover, the PIWIL2 was the target gene of Nrf2, which can repress TGF-β signal transduction by interacting with heat shock protein 90 and lead to the reprogramming of purine metabolism in RILF. PIWI protein MIWI2 was induced and expressed in lung epithelial cells of a murine model infected with *Streptococcus* pneumonia, ultimately increasing the club cells and leukocyte (The Figures are created with BioRender.com)

Growing evidence shows that the piRNAs/PIWI protein plays important roles in the pathogenesis and progression of various respiratory tract diseases, including pneumonia, tuberculosis (TB), asthma, interstitial lung disease (ILD), pulmonary arterial hypertension and lung cancer (Table 1). In addition, scientists have found evidence that supports the differential expression of the piRNAs/PIWI protein between healthy people and patients with respiratory tract diseases (Table 2). Here, we summarize recent studies regarding functions and mechanism of piRNA/PIWI protein in respiratory tract diseases (Fig. 3).

**Table 1:** PiRNA/PIWI complex as a biomarker in respiratory tract diseases

PiRNA	Diseases	Expression	Function	References
PiRNA-L-138	Lung cancer	Up	Directly bond p60-MDM2 to induce apoptosis	[77]
PiRNA-651	Lung cancer	Up	Promoted cells and tumor proliferation and inhibited apoptosis by inducing cyclin D1 and CDK4 expression	[94]
PiRNA-34871	Lung cancer	Up	Correlated with RASSF1C expression, promoted cell proliferation by ATM-AMPK-p53-p21 pathway	[98]
PiRNA-52200	Lung cancer	Up		
PiRNA-35127	Lung cancer	Down		
PiRNA-46545	Lung cancer	Down		
PiRNA-L-163	Lung cancer	Down	Directly bond with p-ERM	[76]
PiRNA-55490	Lung cancer	Down	Inhibited lung cancer cells and tumor proliferation by binding 3ʹUTR of mTOR messenger RNA	[73]
PiRNA-63076	PAH	Up	Increasing the methylation status of the Acadm promoter	[69]
PIWIL2	RILF	Up	Interacted with heat shot protein 90	[74]

**Table 2:** PiRNAs/PIWI complex with differential expression between patients with respiratory tract diseases and healthy people

Groups	Diseases	Origins	Total piRNAs	Differential expression of piRNAs	References
HBE VS NSCL	Lung cancer	Cell culture	555	69	[76]
LUAD VS HC	Lung cancer	DLK1-DIO3 locus in cell	138	5	[92]
LUSC VS HC	Lung cancer	DLK1-DIO3 locus in cell	138	1	[92]
LUAD VS LUSC	Lung cancer	DLK1-DIO3 locus in cell	138	6	[92]
TNonS VS TS	Lung cancer	Lung tissue	–	55	[91]
NNonS VS NS	Lung cancer	Lung tissue	–	49	[91]
PTB VS HC	TB	Human plasma	6200	777	[72]
TB VS LTBItt	TB	Human plasma	–	4	[88]
TB VS ExC	TB	Human plasma	–	2	[88]
TB VS LTBI	TB	Human plasma	–	2	[88]
BSM VS NC	Asthma	Lung tissue	121	5	[75]
LUNG VS BRAIN	Egyptian HPAI (H5N1)	Duck lung tissue and brain tissue	93,598	–	[81]
NOR VS HYP	PAH	PAs of rat	–	2	[69]

### PiRNA/PIWI complex in pneumonia

Pneumonia is the result of pulmonary inflammation in response to pathogens that include viruses, bacteria, and fungi. Thus, pneumonia is the result of host–pathogen interactions in the lung [83]. Moreover, recent studies showed that piRNAs/PIWI protein might contribute to the potential mechanism of pneumonia. The lung is connected to the environment through the bronchus, and HBE cells act as the first line of defense against pathogens and environmental stressors [84]. After exposure to these stress factors, these exposed cells become disordered in endoplasmic reticulum (ER) homeostasis and lead to activation of the unfolded protein response (UPR) pathway. Of these processes, the expression of PIWIL2 and PIWIL4 are significantly increased, causing the mRNA levels of the CCAAT-enhancer-binding protein homologous protein (CHOP) and NOXA to rise [85]. Similarly, a total of 93,598 piRNAs were expressed in the lung and brain of all experimental ducks infected with Egyptian HPAI (H5N1). Although, 90% of piRNAs are expressed at extremely low levels, piRNAs constitute the highest number of expressed sncRNAs [86]. PIWI protein MIWI2 was induced and expressed in lung epithelial cells of a murine model infected with *Streptococcus* pneumonia, ultimately affecting the composition of pulmonary epithelial cells and the innate immunity of the lung [87]. These results show that the piRNAs/PIWI protein may act as a diagnostics biomarker and therapeutic target for pneumonia.

### PiRNA/PIWI complex in asthma

Asthma is mainly characterized by airway hyper responsiveness (AHR) as well as airway inflammation and airway remodeling resulting from nonspecific stimulus in the airway, with involvement of various cells, including airway epithelial cells, eosinophils, neutrophils, T-lymphocytes and mast cells. BSM cells are the effector cells of bronchoconstriction and produce inflammatory mediators and angiogenic factors. A recent study indicated that 5 piRNAs (DQ596390, DQ597484, DQ595186, DQ582264, DQ597347) were differently expressed in BSM cells by statistically analysing the profile between asthmatic patients and healthy subjects [80]. Therefore, piRNA plays a role in the development of asthma, but there is still a lack of comprehensive studies to fully understand the relevant mechanisms of asthma.

### PiRNA/PIWI complex in pulmonary arterial hypertension (PAH)

PAH is a chronic lung disease caused by functional and structural changes in the pulmonary vasculature, leading to right ventricular failure and premature death [88, 89]. Endothelial dysfunction, activation of fibroblasts and proliferation of smooth muscle cells are the main factors in the pathogenesis of PAH [90]. An investigation by Cui Ma et al. observed that piR-63076 regulated cell proliferation through DNA methylation. PiR-rno-63076 antagomir transfection into PASMC decreases the mRNA expression of Acadm by increasing the methylation status of the Acadm promoter [72]. However, the piR-63076 did not follow the gold-standard of piRNAs library preparation, the molecular mechanism of piRNA/PIWI in PAH needs further study.

### PiRNA/PIWI complex in interstitial lung disease (ILD)

ILD is a heterogeneous group of lung diseases characterized by inflammation or fibrosis within the interstitial space, which can be broadly categorized into idiopathic, autoimmune-related, exposure-related (including iatrogenic), interstitial lung diseases with cysts or airspace filling, sarcoidosis, and orphan diseases. Radiation-induced lung injury (RILF) is a subset of exposure-related interstitial lung diseases [91]. RILF, the main complication of radiotherapy among thoracic cancer patients, not only limits the efficacy of radiotherapy but also seriously affects patients' quality of life. A previous study confirmed that the activation of NF-E2-related factor 2 (Nrf2), which is induced by 2-cyano-3, 12-dioxoolean-1, 9-dien-28-oic acid (CDDO-Me), alleviates RILF. Moreover, the PIWIL2 was the target gene of Nrf2, which can repress TGF-β signal transduction by interacting with heat shock protein 90 and triggering ubiquitin-controlled TGF-β receptor degradation and lead to the reprogramming of purine metabolism in WI-38 cells [79]. To date, there are no effective therapies for ILD. Hopefully, the results of relevant studies on piRNA and PIWI protein can be applied to clinical practice.

### PiRNA/PIWI complex in tuberculosis (TB)

TB is an infectious lung disease caused by *Mycobacterium tuberculosis* (Mtb). However, the pathogenesis of TB has not been completely elucidated. Multiple reports have suggested that ncRNAs, such as miRNAs, can play a vital role in the Mtb infection process by acting as diagnostic biomarkers [92, 93]. A previous study explored the differences in piRNA profiles though deep sequencing and real-time PCR (RT-PCR). Based on their previous research, Xing Zhang et al. chose two of four human PIWIL proteins (PIWIL2 and PIWIL4) as symbolic proteins to investigate the activity of piRNA pathways in the peripheral blood of PTB patients by Western blotting. The study found 428 upregulated piRNAs and 349 downregulated piRNAs between healthy people and PTB patients. The sequencing date were verified by RT-PCR, demonstrating the authenticity of the results. Their study indicated that piRNAs had the potential as diagnostic biomarkers for TB. The pathway analyses of transcriptome data indicated that the target genes of differentially expressed piRNAs are involved in cancer-related pathways, such as regulation of the actin cytoskeleton, the Rap1 signaling pathway and the cGMP–PKG signaling pathway. Similar to miRNA, piRNAs can degrade specific mRNAs. According to the Gene Ontology (GO) annotation analysis, the differentially expressed piRNAs might be involved in the process of transcription, regulation of transcription and signal transduction in the biological process (BP) subgroup, but this prediction still requires further validation. Given the potential of these differently expressed piRNAs as diagnostic biomarkers for TB, further studies should be carried out to clarify the mechanisms by which these piRNAs contributes to the pathogenesis of TB, especially in the processes of protein binding, metal ion binding, and ATP binding [77]. Another study on the expression of piRNAs in TB reported that 11 piRNAs were differently expressed between the TB group, the ExC group (exposed controls), the LTBI (latent TB infection) group and the LTBItt group (treated LTBI). PiR-020381 and piR-020490 were identified as moderately accurate biomarkers for LTBI, and piR-009059 was identified in LTBI treatment [94]. The early diagnosis of TB is of vital significance for the treatment of TB patients. The study of piRNA/PIWI protein can serve as a new approach for the diagnosis of TB.

### PiRNA/PIWI complex in lung cancer

Lung cancer is a malignant neoplastic disease with the highest incidence and mortality of all cancers [95]. Some studies have compared differential expression of the piRNAs/PIWI protein in patients with lung cancer and healthy subjects. One study analyzed the expression of piRNAs in the tumor tissues from 3020 patients with hypoxic and non-hypoxic tumors. It identified 33 hypoxia-related piRNAs in adenocarcinomas and 17 hypoxia-related piRNAs in squamous cell carcinomas. In addition, by testing the expression of DQ590404 and DQ596992 in A549 cells with VHL and HIF-1α knockdown, the researchers found that hypoxia-related piRNAs increased via VHL knockdown in a HIF-1α dependent manner [96]. Natasha Andressa Nogueira Jorge et al. discovered that piRNAs were not differentially expressed between normal non-smokers (NNonS) and normal smokers (NS). However, in the lung tissue samples of non-smoking lung cancer patients and smoking lung cancer patients, 55 sncRNAs were differentially expressed, and of these, 2 piRNAs were upregulated (has-piR-010894-3 and has-piR-001168-4) [97]. Previous studies have found that the dysregulation of the DLK1-DIO3 locus on chromosomes 14q32.1-14q32.31 was related to the development of respiratory tract diseases (including cancer). Katey SS Enfield et al. analyzed the piRNAs encoded by the DLK1-DIO3 gene locus among the lung adenocarcinoma cells, lung squamous cell carcinoma cells and normal lung tissues. They found that 7 piRNAs were expressed in three groups, of which 4 piRNAs (DQ596225, DQ596306, DQ596309, DQ596354) were overexpressed in lung adenocarcinoma, and 1 piRNA (DQ596309) was overexpressed in lung squamous cell carcinoma [98]. Through survival curve analysis, intermediate-risk patients could be classified as high-risk patients according to the characteristics of miRNA and piRNA, indicating that piRNA could predict the prognosis of lung cancer patients more precisely. Through piRNA microarray screening, Jia Cheng et al. found that the expression of piRNA-651 increased in both gastrointestinal cancer and lung cancer tissues [99]. Dan Li et al. later studied the mechanism of action of piRNA-651 in non-small cell lung cancer. A xenograft nude mice model was established by injecting A549 cells transfected with the piRNA-651 plasmid, and it was found that the overexpression of piRNA-651 regulates cyclin D1 and cyclin-dependent kinase4 (CDK4), thereby promoting tumor growth [100]. Comparing paired tumors and normal tissues collected from 71 patients with non-small cell lung cancer, Alfons Navarro et al. found that PIWIL1 was expressed in 11 tumor samples but not in normal tissue samples. Patients who expressed PIWIL1 had a shorter disease-free survival than patients who did not express PIWIL1. In addition, compared with normal tissues, the expression of PIWIL2 and PIWIL4 was downregulated in tumor tissues. PIWIL4 levels were directly proportional to tumor recurrence time (TTR) (p = 0.048) and overall survival (OS). Treatment with methyltransferase inhibitor (5ʹ-aza-2-deoxycytidine) and genome bisulfite sequencing analysis showed that the expression of PIWI1 can be partly regulated by methylation [101]. These results indicated that the piRNA pathway can affect the growth of lung cancer and is of great importance in predicting the prognosis of patients. In addition, these results showed that methylation plays a key role in the piRNA pathway. Yuping Mei et al. performed RNA sequencing in HBE cells and non-small cell lung cancer cells. They found a total of 555 piRNAs, of which 51 piRNAs/piRNA-Ls were differently expressed. The ezrin/radixin/moesin (EMR) family is a recently discovered membrane cytoskeleton junction protein family that is expressed on the surface of cell membranes and plays an important role in cell growth, movement, migration, mitosis, and signal transduction. RNA pull down and immunoprecipitation experiments showed that piRNA-L-163 directly bonded to p-ERM and regulated its activity, affecting the proliferation and migration ability of cells [81]. Liping Peng et al. found that the expression of piR-55490 in lung cancer specimens was lower than normal and it suppressed tumor development in lung cancer. Interestingly, piR-55490 binds to the 3ʹUTR of mTOR mRNA and induces the degradation of mTOR mRNA in a manner similar to miRNA [78]. It is worth noting that the expression level of piR-55490 is negatively correlated with patient survival. Dong Liang et al. constructed a plasmid that induced U6 promoter-driven HIWI antagonism. By regularly injecting the plasmid into the tail vein of xenograft tumor mice to observe the growth of xenograft tumors, they found that intravenous injection of the Hiwi shRNA plasmid could significantly inhibit tumor growth [102]. This study aimed to determine whether the strategy of suppressing Hiwi expression based on RNA interference would inhibit tumor growth in xenogeneic mouse models. Yuguang Wang et al. recently proved that HIWI was overexpressed in non-small cell lung cancer tissues and up-regulation of HIWI could promote lung cancer cell proliferation [103]. This study further illustrated that HIWI exerts a carcinogenic effect on lung cancer. These results imply that silencing of the PIWI protein family can be used as a potential treatment option for lung cancer treatment. The RASSF1C gene can promote the growth of lung cancer cells. Two upregulated piRNAs (piR-34871 and piR-52200) and two down-regulated piRNAs (piR-35127 and piR-46545) were found through piRNA microarray analysis of lung cancer cells with overexpressed RASSF1C and silencing of RASSF1C. This study showed that overexpression of piR-35127 and piR-46545 or knockout of piR-34871 and piR-52200 could promote the proliferation of lung cancer cells [104]. RRBS showed that RASSF1 and PIWIL1 could regulate gene DNA methylation. Knockdown of RASSF1 and PIWIL1 increased the expression of GMIP (a hypermethylated gene), which, in turn, caused the proliferation and migration of lung cancer cells. The RASSF1C-PIWIL1-piRNA pathway promoted the proliferation and migration of lung cancer cells by regulating DNA methylation [71]. After treating lung squamous cells with four different chemotherapy drugs, Yuyan Wang et al. found that the expression of piR-L-138 increased significantly in the four groups. Moreover, in the knockdown of piR-L-138, the cell viability of lung squamous cells decreased and apoptosis increased. A study showed that in cisplatin-treated lung squamous cell carcinoma cells, piR-L-138 directly bound p60-MDM2 to induce apoptosis [81]. This study provided a new strategy for lung cancer patients to overcome chemical tolerance, piRNA should be applied in clinical practice as soon as possible for the benefit of patients.

## Conclusion

PiRNA and the PIWI protein are associated with the initiation and development of several respiratory tract diseases, especially lung cancer, primarily through TGS and PTGS. The studies described in this review can provide new ideas on how to improve the efficiency and convenience of the diagnosis and treatment of these respiratory tract diseases. At present, piRNA-related drugs for treatment have not yet been discovered, as studies about the piRNA/PIWI complex in respiratory tract diseases are limited, mainly in basic research of cancers. However, the piRNA/PIWI complex have the potential to become a diagnostic biomarker and therapeutic target in the clinic following the development of technologies involving molecular targeted therapy.

## Data Availability

Not applicable.

## Abbreviations

Acadm: Acly-CoA dehydrogenase
AGO: Argonaute proteins
AHR: Airway hyper responsiveness
Arx: Asterix protein
Aub: Aubergine
BP: Biological process subgroup
BSM: Bronchial smooth muscle cells
CCR4-NOT: Carbon catabolite-repressed 4-negative on TATA-less
CDK4: Cyclin-dependent kinase 4
CDDP: Cisplatin
CHOP: CCAAT-enhancer-binding protein homologous protein
DE: Differentially expressed
Egg: Eggless
EMR: Ezrin/radixin/moesin
ExC: Exposed controls group
Gasz: Germ cell protein with Ankyrin repeats, Sterile alpha motif, and leucine Zipper
GMIP: Methylated Gen interacting protein
GO: Gene Ontology
HBE: Human bronchial epithelial cells
HC: Healthy individuals
HIWI: The human homolog of the PIWI family
HPAI: Highly pathogenic avian influenza
HP1: Heterochromation protein 1
HS: Healthy subjects
HYP: Hypoxic rats
H3K9me2: Histone 3 lysine 9 dimethylation
H3K4me2: Histone 3 lysine 4 dimethylation
H3K9me3: Histone 3 lysine 9 trimethylation
H5N1: Hemagglutinin 5 neuraminidase 1
ILD: Interstitial lung disease
LncRNA: Long noncoding RNA
LSCC: Lung squamous cell carcinoma
LTBI: Latent TB infection group
LTBItt: Treated latent TB infection group
LUAD: The lung adenocarcinoma cells
LUSC: The lung squamous cell carcinoma cells
Mino: Minotaur
Mtb: Mycobacterium tuberculosis
mRNA: Messenger RNA
miRNA: MicroRNA
MIWI2: Murine PIWI protein 2
mTOR: Mammalian target of rapamycin
NcRNA: Non-coding RNA
NC: Normal lung tissues
NHBE: Normal HBE cells
Nrf2: NF-E2-related factor 2
NNonS: Normal non-smoker
NS: Normal smoker
NOR: Normoxic rats
NSCL: Non-small cell lung cancer
OS: Overall survival
PAH: Pulmonary arterial hypertension
PAs: Pulmonary arteries
PASMC: Pulmonary arterial smooth muscle cells
Panx: Panoramix
PIWI: P-element-induced wimpy testes protein
piRNA: PIWI-interacting RNA
PIWIL2: PIWI-like RNA-meditate gene silencing 2
PIWIL4: PIWI orthologs 4
PiR-L-163: PiRNA-like-163
PTGS: Post-transcriptional gene silencing
PTB: Pulmonary tuberculosis
P60-MDM2: Mouse doubleminute 2 homolog
RILF: Radiation-induced lung fibrosis
RISC: RNA-induced silencing complex
RRBS: Reduced representation bisulfite sequencing
RT-PCR: Real-time PCR
TB: Tuberculosis
TGS: Transcriptional gene silencing
Smg: Smaug
TNonS: Tumor non-smoker
TS: Tumor smoker
TTR: Time to recurrence
Ste: Stellate
Su (Ste): Suppressor of Stellate
UPR: The unfolded protein response pathway
Vert: Vreteno
Wde: Windei
ZUC: Zucchini
